# Diagnostic Value of Machine Learning-Based Quantitative Texture Analysis in Differentiating Benign and Malignant Thyroid Nodules

**DOI:** 10.1155/2019/6328329

**Published:** 2019-10-31

**Authors:** Bulent Colakoglu, Deniz Alis, Mert Yergin

**Affiliations:** ^1^Vehbi Koç Foundation American Hospital, Department of Radiology, Istanbul, Turkey; ^2^Istanbul Mehmet Akif Ersoy Thoracic and Cardiovascular Surgery Training and Research Hospital, Department of Radiology, Halkali, Istanbul, Turkey; ^3^Bahcesehir University, Department of Software Engineering and Applied Sciences, Istanbul, Turkey

## Abstract

**Aim:**

The aim of this study is to evaluate the diagnostic value of machine learning- (ML-) based quantitative texture analysis in the differentiation of benign and malignant thyroid nodules.

**Materials and methods:**

A sum of 306 quantitative textural features of 235 thyroid nodules (102 malignant, 43.4%; 133 benign, 56.4%) of a total of 198 patients were investigated using the random forest ML classifier. Feature selection and dimension reduction were conducted using reproducibility testing and a wrapper method. The diagnostic accuracy, sensitivity, specificity, and area under curve (AUC) of the proposed method were compared with the histopathological or cytopathological findings as reference methods.

**Results:**

Of the 306 initial texture features, 284 (92.2%) showed good reproducibility (intraclass correlation ≥0.80). The random forest classifier accurately identified 87 out of 102 malignant thyroid nodules and 117 out of 133 benign thyroid nodules, which is a diagnostic sensitivity of 85.2%, specificity of 87.9%, and accuracy of 86.8%. The AUC of the model was 0.92.

**Conclusions:**

Quantitative textural analysis of thyroid nodules using ML classification can accurately discriminate benign and malignant thyroid nodules. Our findings should be validated by multicenter prospective studies using completely independent external data.

## 1. Introduction

Thyroid nodules are common, with a prevalence of up to 67% in the adult population [1, 2]. Approximately 5%–15% of these nodules are malignant, and the differentiation of malignant and benign nodules is mandatory for forming individual management strategies [3–7]. Ultrasound (US) is the first and most commonly used imaging modality for the evaluation of thyroid nodules [2, 3]. The nodules that are strongly suspected to be malignant as appraised by US are further evaluated by fine-needle aspiration biopsy (FNAB) or tissue biopsies; hence, a noninvasive method with an ability to differentiate malignant and benign nodules is desirable [8, 9]. Sonographic features such as irregular margin, solid composition, hypoechogenicity, elongated shape, and microcalcifications indicate malignancy [8, 9]. However, these features are somewhat qualitative, and the experience of a radiologist has a substantial influence on diagnostic accuracy [10–15]. Over the years, various reporting systems have been introduced to reduce inconsistencies among radiologists and promote communication between clinicians and radiologists. However, these reporting systems still rely on a radiologist's subjective interpretations. Moreover, some radiologists are reluctant to use these reporting systems owing to their complexity [8–10].

Radiomics is defined as the machine learning- (ML-) or deep learning-based mining of quantitative texture features extracted from conventional imaging modalities. The aim is to improve the precision and diagnostic accuracy of imaging methods, mostly in the field of cancer research [16, 17]. Several studies have demonstrated the feasibility of ML-based texture features in differentiating between benign and malignant thyroid nodules [18–28]. Nevertheless, ML-based texture analysis for the evaluation of thyroid nodules is still in its infancy, and further research is necessitated.

Herein, we evaluate the diagnostic performance of ML-based quantitative texture analysis in differentiating malignant and benign thyroid nodules.

## 2. Materials and Methods

The local ethics committee approved this retrospective study, which was conducted between January 2015 and January 2019. We reviewed our picture archive and communicating system to identify patients who underwent thyroid US examination for thyroid nodules. The patients in whom the thyroid nodules were determined as benign or malignant according to FNAB or surgical pathology were included in the study. Nodules that fell into the nondiagnostic or indeterminate categories according to the Bethesda Classification System and thyroid nodules <1 cm in diameter were excluded from the study [4]. All of the nodules were evaluated by the same radiologist (B.C.), who had more than 20 years of experience in thyroid US, with the same device (LOGIQ E9 with XDclear, General Electric (GE) Healthcare, Wauwatosa, WI, USA) using a linear array transducer (ML6-15) with a frequency range of 12 to 15 MHz. Grayscale images of the thyroid nodules in the axial plane were selected for further analysis.

### 2.1. Texture Feature Extraction

US images contain inherent impulse and salt-and-pepper type noise; hence, an anisotropic median filter was applied to all US images before the texture extraction step to remove noise while preventing the edges and boundaries of images being blurred. QMaZda texture analysis software was used for quantitative texture feature extraction [29]. The radiologists manually delineated the borders of the thyroid nodules for texture extraction. The image histogram was remapped within ±3*σ* of the grayscale levels to prevent texture features from being affected by image characteristics such as contrast or brightness [29].

A total of 306 texture features were extracted for further analysis [28]: first-order histograms (13), gradient-map-based features (5), gray-level co-occurrence matrix (GLCM) features (176), gray-level run-length matrix (GRLM) features (28), autoregressive model features (5), Haar wavelets (12), Gabor transform features (24), histogram of oriented gradients (HOG; 8), and local binary patterns (LBP; 35) [30]. GLCM and GRLM were calculated at 5 bits per pixel, and gradient-map-based features were calculated at 4 bits per pixel. First-order histograms, autoregressive model features, and Haar wavelet features were calculated at 8 bits per pixel. LBPs were calculated by one of the three algorithms:over-complete (Oc), transition (Tr), and center-symmetric (Cs),with respect to the number of 4*n* neighbors. The extracted texture features were further entered into ML-based analyses.

### 2.2. Feature Selection and Dimension Reduction

Waikato Environment for Knowledge Analysis (WEKA) toolkit version 3.8.2 (University of Waikato, Hamilton, New Zealand) was used for feature selection [31]. The current study, similar with most other studies involving quantitative texture analysis, had a substantially higher number of texture features (*n* = 306) than thyroid nodules in the study cohort (*n* = 235). This is a recognized problem in ML analysis and is also known as the curse of dimensionality. As a consequence, there is a significant risk of overfitting the model. Over-fitted models can be briefly described as models that strictly fit the training data but show poor performance on new cases, namely, the test set. Hence, there is a wide agreement as to the importance of feature selection before creating the ML model for classification or prediction tasks. To detect the most appropriate features while discarding irrelevant ones for the model, we employed three consecutive steps. First, two radiologists (B.C. and D.A.) drew regions of interest onto randomly selected images of benign and malignant thyroid nodules (10 images each) to assess the reproducibility of the extracted texture features. Features with a good intraclass correlation (ICC) value (≥0.80) were further considered in the following steps. Next, a scheme-dependent feature selection method, a wrapper subset evaluation using 10-fold cross-validation, was applied [31, 32].

**Figure 1:**
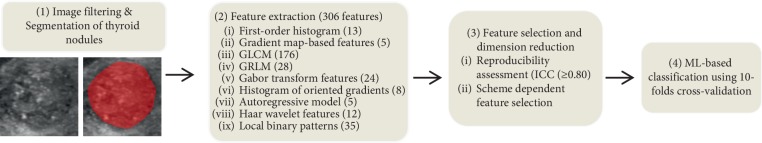
The scheme summarized the main workflow of the current study.

A wrapper method is a supervised scheme-dependent feature selection technique that evaluates the features according to their importance to the model [32]. The wrapper method evaluates the relevance of the attributes based on a classifier, which was the random forest classifier in the present work. The wrapper method first creates multiple subsets of the features and then tests the performance of these subsets to find the best combination of features. There are several types of wrapper methods depending on the search method. The current study uses the wrapper method with linear forward stepwise selection, in which the search for the most relevant features for the model begins with a null model and continues until the best subset of the attributes are determined [32]. We applied feature selection after cross validation; hence, relevant features were selected using only the training partitions of the dataset to avoid the “double-dipping” phenomenon, which occurs when the whole dataset is used for the selection and might lead to biased or over-optimistic results [32, 33]. The selected features were further processed using the ML classifier (a random forest) to assess its diagnostic performance in discriminating benign and malignant thyroid nodules.  Figure 1 summarizes the pipeline of the present work.

### 2.3. ML Models and Statistical Analyses

**Figure 2:**
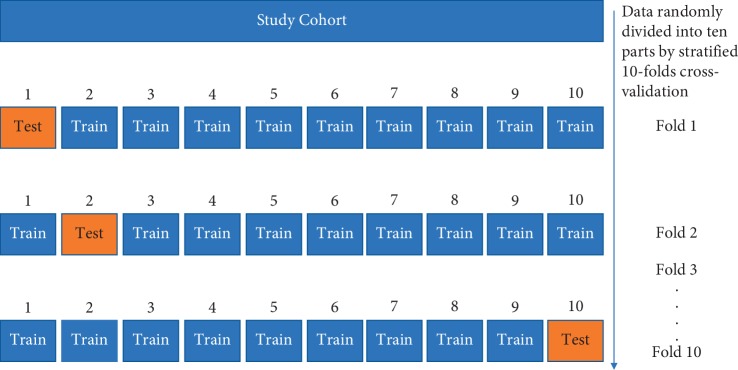
Evaluation of the model's performance by 10-fold cross-validation. 10-fold cross-validation first randomly divides all the data into ten parts then holds out 10% of the data for testing. This process is repeated ten times, and then the mean accuracy for the algorithm is calculated.

WEKA toolkit version 3.8.2 was used to develop the ML model and to test its performance. Only selected quantitative texture features were used in the ML analysis. The present study used the random forest classifier to build the ML model. The random forest is a well-known ML algorithm for classification tasks and has an inherent resistance to overfitting [34]. The random forest is an ensemble learning method. It chooses random data points from the dataset to build multiple decision trees and then uses all of these decision trees to improve the performance of the final prediction [31]. We did not split the cohort into training and testing groups; instead, we applied stratified 10-fold cross-validation, which randomly divides all the data into ten parts and then holds out 10% of the data for testing. This process is repeated ten times [35]. A detailed illustration of the 10-fold cross-validation method is given in Figure 2.

The diagnostic performance of the ML model was assessed using correlation matrices, which shows the results as the number of true positives (TP), true negatives (TN), false positives (FP), and false negatives (FN) according to the histopathological sampling results. The following formulas were then used to determine performance: sensitivity = TP/(TP + FN), specificity = TN/(TN + FP), and diagnostic accuracy = (TP + TN)/(TP + TN + FP + FN). The receiver operating curve was drawn for the ML model and the area under the curve (AUC) was calculated. The wrapper method is able to identify the best subset for the ML model, but it does not provide further information regarding the relative importance of the selected features. Hence, we employed an information gain attribute evaluator, which evaluates the worth of an attribute (in this case, the discriminative power of the attributes for benign and malignant thyroid nodules) by measuring the information gain with respect to the class [31]. The following formula was used for the information gain attribute evaluator: InfoGain(Class, Attribute) = *H*(Class) − *H*(Class | Attribute), where *H* represents the amount of information in a unit called bits and ranges in value between 0 and 1 [31]. The information value increases as the value approaches 1.

## 3. Results

The final cohort study comprised a total of 235 thyroid nodules of 198 patients (150 females, 48 males; age range 18–81 years; and mean age 44.55 years). There were 98 patients with 102 malignant thyroid nodules and 100 patients with 133 benign thyroid nodules. Of the 98 patients with malignant thyroid nodules, 33 were male and 65 were female with a mean age of 42.12 ± 14.55 years. Of the 100 patients with benign thyroid nodules, 22 were male and 78 were female with a mean age of 46.35 ± 17.12 years. Among the 102 malignant nodules, the FNAB results of 82 nodules (80.3%) were also confirmed by surgical pathology as 73 papillary thyroid carcinomas (89%) and nine follicular variants of papillary cancer (11%). The other malignant thyroid nodules (*n* = 20) had only a cytopathological diagnosis because the patients did not undergo an operation at our institution. Of the 306 initial texture features, 284 (92.2%) showed good reproducibility (ICC ≥ 0.80) and were further used for the ML-based evaluation. A total of seven texture features were selected for the final model: one histogram (HistPerc 99), one HOG (HogO8b2), four GRLM (GrlmHRLNonUni, GrlmHMGLevNonUni, GrlmNRLNonUni, and GrlmZRLNonUni), and one GLCM (GlcmZ3AngScMom).

HistPerc 99 is an alternative to maximum intensity that is equal to the highest intensity in the defined region of interest [36, 37]. The mean HistPerc 99 values of the benign nodules were higher than those of the malignant ones in the present study, indicating the higher intensity values in benign tumors. The GRLM counts the runs of pixels with the same gray level in different directions [36, 37]. The selected GRLM features were the following in the present work: GrlmHRLNonUni, GrlmHMGLevNonUni, GrlmNRLNonUni, and GrlmZRLNonUni. In the feature names, “Grlm” represents GRLM, the letters “H,” “N,” and “Z” represent the direction of that feature: *H* = 0° (horizontal), *Z* = 45°, and *N* = 135° [29]. Furthermore, MGLevNonUni and RLNonUni represent nonuniformity, in which a higher value indicates heterogeneity [29, 36, 37]. In the present work, malignant thyroid nodules had more runs with nonuniform values, indicating the heterogeneity of the nodules. GLCM features count the number of co-occurrences of pixels with specified gray-levels. Pairs of pixels are considered, such that one of the pixels is situated at an offset (Δ*x*, Δ*y*) with respect to the other [36, 37]. The random forest model used GlcmZ3AngScMom, where “Glcm” represents the GLCM, “Z3” represents the direction (45°) and the offset of the pixels, and AngScMom represents angular second movement and also energy, which is a measure of homogeneous patterns in the image [29, 36, 37]. Benign thyroid nodules had higher mean GlcmZ3AngScMom values in the present work. The HOG counts the number of occurrences of gradient. It identifies a sudden change in the pixel values, which is called the gradient [29, 36, 37]. A positive gradient refers to a change from a lower to higher pixel value while a negative gradient refers to a higher-to-lower change in value. HOGs are also a known marker of heterogeneity. In the present work, malignant nodules had higher gradient reflecting the heterogeneity of the tumor.

**Figure 3:**
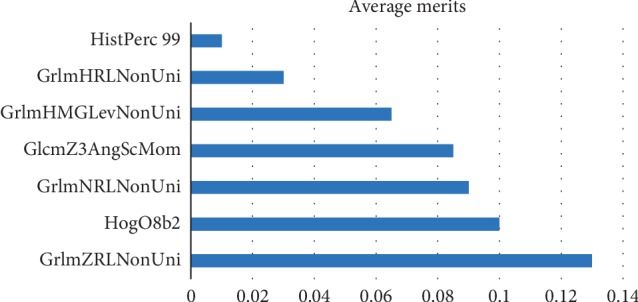
A total of seven texture features were selected for the final model: one histogram (HistPerc 99), one HOG (HogO8b2), four GRLM (GrlmHRLNonUni, GrlmHMGLevNonUni, GrlmNRLNonUni, and GrlmZRLNonUni), and one GLCM (GlcmZ3AngScMom). The information gain attribute evaluator identified that GrlmZRLNonuni was the most important feature in the final model followed by HogO8b2 and GrlmNRLNonUni. The formula of the information gain attribute evaluator was InfoGain(Class, Attribute) = *H*(Class) − *H*(Class | Attribute), where *H* represents the amount of information in a unit called bits and ranges in value between 0 and 1.

The random forest classifier accurately identified 87 of the 102 malignant thyroid nodules and 117 of the 133 benign thyroid nodules. These values equate to a diagnostic sensitivity of 85.2%, a specificity of 87.9%, and an accuracy of 86.8%. The AUC was calculated as 0.92 for the model. The average values of the quantitative texture features for the differentiation of malignant and benign thyroid nodules are shown in Figure 3.

## 4. Discussion

The present study demonstrated that the ML model using the random forest classifiers with selected texture features can successfully discriminate malignant and benign thyroid nodules. The selected texture features of the random forest model consisted of histogram, HOG, GRLM, and GLCM features. The selected second-order features mainly reflect the increased heterogeneity of the malignant thyroid nodules, whereas the histogram feature represents the hypoechogenic characteristic of the malignant nodules.

### 4.1. Related Work

Several authors have evaluated the diagnostic value of ML-based texture analysis for the differentiation of benign and malignant thyroid nodules. The diagnostic accuracy has even reached 100% in some of these works. For instance, Acharya et al. [19–21] demonstrated that ML-based texture analysis had a diagnostic accuracy ranging from 98.3% to 100%, but the study cohorts consisted of only 20 nodules in three of their reports. In the work by Chang et al. [22], the support vector machines classifier showed a diagnostic accuracy reaching up to 98.3% for 59 nodules. A recent work by Prochazka et al. [38] employed a random forest and support vector machine classifier for evaluating segmentation-based fractal texture analysis, and the authors achieved a diagnostic accuracy of 94.3% with their model. Although all studies mentioned above have yielded promising results, the use of a small sample size in ML-based diagnostic models will undoubtedly introduce bias and variance [39]. Furthermore, using such small cohorts increases the risk of overfitting and limits the generalizability of the results [39].

To our knowledge, there are few other studies on the ML-based quantitative texture analysis of thyroid nodules that have a cohort size that is comparable to the size of the one in our work. These works reported diagnostic accuracies ranging from 78.5% to 94.3%, which is comparable with the diagnostic accuracy of 86.8% obtained by the random forest in the present work [23, 26, 28]. Song et al. [26] evaluated 16 GLCM features using logistic regression, artificial neural network, random forest, boost, SVM, and random tree models. They found that the logistic regression model achieved the highest diagnostic accuracy. Similar to our work, they did not use different data for training and testing; instead, they implemented 10-fold cross-validation [26]. Acharya et al. [23] evaluated the Gabor transform features of 242 benign and malignant thyroid nodules using support vector machines, *k*-nearest neighbors, multilayer perceptron, and C4.5 decision tree classifiers. In their work, the C4.5 decision tree classifier achieved the best diagnostic performance with a diagnostic accuracy of 94.3%. Yu et al. [28] implemented artificial neural network classifiers for the evaluation of two morphological and 65 different texture features. They trained the initial models using images of 610 thyroid nodules with 10-fold cross-validation and achieved 99% diagnostic accuracy. They externally validated their model using images of 50 thyroid nodules, which resulted in a diagnostic accuracy of 90% [28]. We suggest that the better accuracy obtained by Yu et al. [28] might be a consequence of the inclusion of semantic parameters such as the orientation and boundaries of the nodules. It is well known that semantic features such as vertical shape and irregular borders are closely associated with malignancy; hence, they might improve the ML-based models.

### 4.2. Limitations

First and foremost, there is an inevitable selection bias in the present work because we only included thyroid nodules categorized as benign or malignant according to FNAB or surgical pathology; hence, we excluded most of the benign nodules that did not have biopsy results. In daily practice, a relatively small number of thyroid nodules are scheduled for histopathological examination, and most of the thyroid nodule data with benign features were follow-ups using US [3, 4]. Therefore, benign thyroid nodules in the present work might not cover all types of benign nodules. Second, we did not evaluate the semantic features of thyroid nodules nor integrate qualitative US features such as echogenicity or nodule composition because our aim was to assess the diagnostic value of textural analysis alone. Third, we neither compared the diagnostic accuracy of our ML model with human evaluators nor evaluated the diagnostic accuracy of a human evaluator supplemented by the ML model. Hence, we suggest that further studies investigating the diagnostic accuracies of human evaluators and ML-based classifications as well as the assistive value of ML-based models for human evaluators might be worthwhile. Fourth, we did not use separate test and training groups; instead, we implemented the 10-fold cross-validation algorithm, which allows us to use the same cohort as test and training subjects [35]. Finally, although it is not peculiar to the present work, to date, many ML-based classification systems and an abundant number of different texture features have been evaluated for differentiating thyroid nodules as benign or malignant. Hence, the standardization of the models and evaluated features is a concerning issue that prevents the use of ML models for the characterization of thyroid nodules in practice [40].

## 5. Conclusion

We demonstrated that an ML classifier, the random forest, with selected textural features can achieve 85.2% sensitivity, 87.9% specificity, and 86.8% diagnostic accuracy with an AUC of 0.92 in the task of differentiating malignant thyroid nodules from benign ones. The texture features selected in this study indicate that malignant thyroid nodules have increased heterogenicity and lower echogenicity than benign thyroid nodules. We acknowledge that our findings should be validated by prospective multicenter studies using a completely independent external dataset.

## Data Availability

The data supporting our findings can be found in the article. Given the strict regulations of our institutional review board, further data of the patients are only available from the corresponding author on reasonable request.

## Abbreviations

AUC: Area under the curve
GLCM: Gray-level co-occurrence matrix
GRLM: Gray-level run-length matrix
HOG: Histogram of oriented gradients
ML: Machine learning
US: Ultrasound
WEKA: Waikato Environment for Knowledge Analysis.
